# Decoding communicative action vitality forms in social contexts

**DOI:** 10.3389/fpsyg.2025.1478875

**Published:** 2025-02-05

**Authors:** Radoslaw Niewiadomski, Amrita Suresh, Alessandra Sciutti, Giuseppe Di Cesare

**Affiliations:** ^1^Department of Informatics, Bioengineering, Robotics, and Systems Engineering, University of Genoa, Genoa, Italy; ^2^Cognitive Architecture for Collaborative Technologies Unit, Italian Institute of Technology, Genoa, Italy; ^3^Robotics Research Group, Faculty of Mathematics and Computer Science, University of Bremen, Bremen, Germany; ^4^Department of Medicine and Surgery, University of Parma, Parma, Italy

**Keywords:** social interaction, action - investigation, human interactions, vitality affects, affective communication

## Abstract

The form of an action conveys important information about the agent performing it. Humans may execute the same action in different ways, e.g., vigorously, gently or rudely. This fundamental affective component of the action has been named vitality forms (VFs) by Stern. To date, despite the fundamental role of VFs in social communication, the kinematic features characterizing them have been rarely studied. The aims of the present study are twofold: to investigate spatiotemporal characteristics of transitive and intransitive actions performed with different VFs; to investigate whether and how it is possible to recognize these aspects of action automatically. For this purpose, we asked two actors to perform seven actions with VFs (gentle and rude) or without VFs (neutral, slow and fast). Thousand repetitions of actions were collected, and their kinematics was recorded by using a motion capture system. Twenty-two kinematic features were identified from kinematic data. Results indicate that VFs are not merely characterized by a modulation of a single motion parameter such as velocity or acceleration, but by a combination of different spatiotemporal properties. Finally, we also demonstrate that automatic recognition of VFs is possible using traditional machine learning methods, with an accuracy of 87.3%. Moreover, this recognition is also feasible for action types do not present in the training set, with an accuracy of 74.2%. These results will have significant implications in the future across various fields, including neuroscience, social robotics, and the development of virtual agents. For instance, it could enable artificial agents to recognize human attitudes and adapt their behavior appropriately to the partner during interactions. Moreover, understanding the VFs features could be useful in designing rehabilitative interventions for conditions involving social and communicative impairments, such as autism.

## Introduction

Social communication requires the ability to correctly interpret people’s nonverbal behaviors (e.g., gestures) and to timely anticipate others’ actions. While interacting with other individuals, we can understand their attitudes as well as their intentions. In addition to the goal (what) and motor intention (why), human actions are also characterized by the form (how). Indeed, the same action can be performed in different ways, for example gently, excitedly, vigorously or rudely communicating our attitudes toward others. These forms of action have been named vitality forms (VFs) by Stern (2010). According to Stern, VFs reflect the internal psychological state of the agent, providing also an appraisal of the affective quality underlying the relation between the agent and the action recipient. Moreover, VFs characterize all human actions and are detected based on certain movement dynamics: time profile (start, duration and the end of an action), force, space and direction (Stern, 2010). VFs describe the affective component of an action. Identifying VFs of an action allows the observer to infer the agent’s affective state as well as their relationship with the action recipient. For instance, a kind or rude action may reveal whether the agent’s mood is negative or positive. The ability to communicate and understand VFs emerges early in infancy, highlighting their importance for developing social attunement (Condon and Sander, 1974; Nadel and Butterworth, 1999; Flom and Bahrick, 2007). These capacities represent a primordial way of relating to and understanding others and are likely a foundational component of interpersonal relationships (Trevarthen, 1998; Gallese and Rochat, 2018; Liu et al., 2022). It is important to note that VFs can be communicated through more than one modality: by observing gestures (Di Cesare et al., 2014), voice intonation (Di Cesare et al., 2022), and even touch (Rizzolatti et al., 2021). It has been also shown that VFs can also be automatically detected from videos of gestures (Canovi et al., 2024) as well as touch actions (Niewiadomski et al., 2023).

VFs differ from emotions, and especially from basic emotions, in several respects, such as in triggering factors, duration and the voluntary nature. Basic emotions (such as joy, anger, sadness, surprise, disgust and fear) are short-lasting events triggered by internal or external factors, typically ending soon after the stimuli cease. The emotions usually are not voluntary, that is, they *typically* arise spontaneously and are not consciously controlled, emerging as responses to specific stimuli or situations rather than being the result of deliberate choice or intentional effort. The emotions are not necessarily related or expressed by specific actions (e.g., one may feel angry without performing any hand movement) and very often induce visceromotor responses (James, 1884). In contrast, VFs are voluntary events that are expressed continuously. They can be influenced by external factors like the social context (Stern, 2010). For example, based on our positive or negative mood or attitude, we can greet a friend warmly or coldly. This is completely in line with Køppe et al. (2008) who reported that VFs are qualitatively distinct from classical Darwinian emotions. Indeed, unlike emotions, VFs are broadly connected to fundamental life processes such as breathing, hunger, sleep, and waking, which embody the essence of ‘vitality’. Moreover, VFs are also intrinsically present in goal-directed mental activities, such as the progression of thoughts, social interactions, or dialogs.

VFs represent a fundamental aspect of human interaction and the ability to express and recognize these forms of communication allows people to be socially connected. Indeed, during interpersonal relations, VFs contribute to social communication by modulating gestures or words, while the perception of VFs allows the observer to understand the attitudes of others. Extensive research in neuroscience has provided strong evidence of the neural bases of the generation and comprehension of VFs in humans. In a pioneering functional magnetic resonance imaging (fMRI) study, Di Cesare et al. (2014) demonstrated that when participants paid attention to a goal directed actions, the observation of the action goal produced the activation of the parieto-frontal circuit while when they paid attention to the VFs of the same actions, this produced the activation of the same circuit plus a new area named the dorso-central insula (DCI). These data clearly demonstrated that when observing an action performed by another individual, the encoding of its components (content and form) involves the activation of different brain networks. This view is in line with dynamic causal modeling results of a very recent fMRI study (Di Cesare et al., 2024), which demonstrated that when participants performed a passing action with VFs (gentle, rude) or without VFs (neutral action) there was an initial activation of the premotor cortex (PM) and then two streams originated: one toward the inferior parietal lobule (IPL) and one toward DCI. While the activation of the parieto-frontal areas was common to all performed actions, the activation of the second stream directed from PM to DCI was only present when participants voluntary decided to execute the same action with gentle or rude VFs.

The selective activation of DCI in relation to VFs processing is corroborated by a series of fMRI studies adopting different tasks such as observation of VFs (Di Cesare et al., 2015, 2021), as well as listening to action verbs (Di Cesare et al., 2016) or words (Di Cesare et al., 2022) pronounced with different VFs. Interestingly the same area is also active during the expression of VFs, e.g., the execution of actions (or the pronunciation of words) conveying the same VFs (Di Cesare et al., 2015, 2021). Taken together, these findings suggest the existence of a mirror mechanism for VFs located in the dorso-central insula. It is important to note that also the observation of humanoid robots performing gestures characterized by human-inspired VFs can evoke similar neural responses in the dorso-central area of the observer (Di Cesare et al., 2020). This phenomenon has also behavioral consequences. Indeed, some studies demonstrated that, positive or negative VFs expressed by a human (Di Cesare et al., 2017a; Lombardi et al., 2021) or humanoid agent (Vannucci et al., 2024, 2018; Lombardi et al., 2024), modulated the response of the receiver in terms of kinematics. Specifically, when the agent’s request was rude participants performed a subsequent action with a higher velocity peak, higher trajectory, and a longer distance. In contrast, when the request was gentle the same action was performed with a lower velocity peak, lower trajectory and a shorter distance. All these findings indicated the fundamental role of VFs for interpersonal relations not only between humans, but also between humans and artificial agents.

Despite the importance of VFs in social communication, the physical features characterizing them are poorly understood. To fill this gap the present study aims to: (1) identify the spatiotemporal features that may reveal VFs information of the performed actions; (2) assess whether it is possible to recognize VFs of these actions automatically. To this purpose, by using a motion tracking system, we recorded the 3D positional data of two actors’ hand performing seven goal directed actions with VFs (gentle, rude) and without VFs (slow, fast). Beside these four conditions, at the beginning of the data collection section, we also recorded a fifth condition in which the same actions were performed in a neutral way. We hypothesize that a singular kinematic feature such as the velocity is not sufficient to distinguish between actions performed with and without VFs (e.g., slow actions *versus* gentle actions, fast actions *versus* rude actions). Rather, the combination of several features could be essential for this distinction. This hypothesis is in line with the idea of Stern who considered VFs as a Gestalt created by 5 fundamental dynamic components: movement, time profile, force, space intention and directionality (Stern, 2010).

In the future, the results of this study may contribute to other research domains. For example, understanding the kinematic features of different VFs would be essential for enabling artificial agents (humanoid robots or virtual agents) to recognize and express VFs and, consequently, making these agents behave more naturally and improving their interactions with humans. Indeed, a better understanding of the partner’s VFs will be helpful to plan the correct response (e.g., to show politeness) toward them. Moreover, VFs models may have important applications also beyond human-machine interaction. In future, such computational models can be used as a part of training dedicated to, e.g., neurodivergent individuals who showed reduced ability to correctly perceive and communicate attitudes through VFs (Rochat et al., 2013; Di Cesare et al., 2017a; Casartelli et al., 2020; Di Cesare et al., 2024).

## Materials and methods

**Figure 1:**
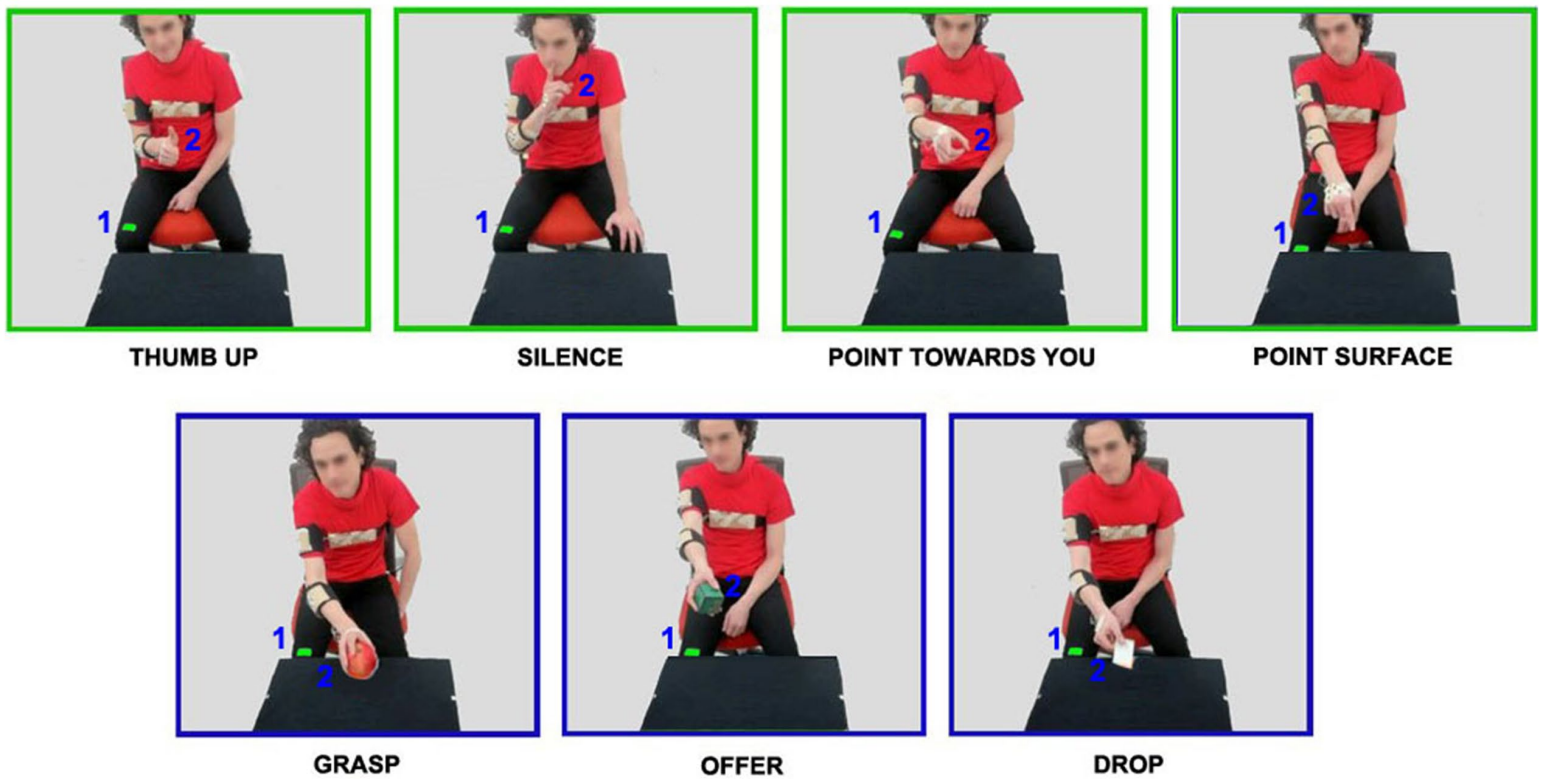
Video frames showing intransitive (green edge) and transitive (blue edge) actions performed by the male actor toward the experimenter sitting in front of him. For each action, the starting position (green cue placed on the right leg) and the ending position are indicated with numbers 1 and 2, respectively.

Two theater actors (1 male, 1 female) were asked to perform with their dominant right hand seven different actions (ethical committee approval of Liguria Region, n.222REG2015). Specifically, three actions were performed with objects (grasping; offering; dropping a game card) and four actions were performed without objects (indicating a point of the surface; thumbing up; rising a finger to the mouth in a silence gesture; pointing toward the other person, Figure 1). All these actions were performed in a social context: toward another person (the experimenter) sitting in front of the actor, who did not perform any specific movement.

For each action type, the hand’s starting and ending positions were predefined by a specific point (starting position: point 1; ending position: point 2; Figure 1). Each session was organized in 3 steps. In the first step, we asked actors to perform all the 7 actions without any VFs. Specifically, during this phase, actors performed each action in a neutral way without receiving any instruction regarding the VFs (baseline). In the second step, both actors were asked to perform the same actions slowly and quickly. In steps 1 and 2, each action was repeated at least 10 times. Finally, in the third step, we introduced actors to the concept of VFs by presenting video-clips showing two individuals performing transitive (passing a bottle, offering a packet of crackers) and intransitive actions (stop gesture, caressing) with a gentle and rude VFs. This procedure allowed the actors to focus on the action VFs. Most importantly, during this step, VFs were presented without labels (gentle, rude) to avoid the possibility that the linguistic interpretation of terms could influence the expression of VFs when performed by the two actors. When watching videos, actors were asked to focus on the attitude of individuals (their internal state) and not just on the kinematic features, with the intent to reproduce these attitudes rather than replicating the motion when performing their actions. The choice to use gentle and rude VFs was due to maximize possible differences identifying the kinematic features characterizing them. After this training, each actor was asked to perform the seven actions toward a person sitting in front of him/her, expressing the two VFs observed in the previous videos. Specifically, each actor performed actions gently or rudely (vitality forms condition). To augment the movement variability in the dataset, actors were additionally asked to perform these actions in three different directions: not only toward a sitting person (0 degrees), but also directed to the right side (45 degrees condition), and the left side of the receiver (−45 degrees condition). Considering seven action types, five action conditions (gentle, neural, rude, slow, fast) and two actors, in total 1,051 actions were recorded. Thirty actions (2.9%) were discarded from the dataset due to technical problems (for details see Table 1).

**Table 1:** Number of repetitions performed per each action type and class.

	Rude	Neutral	Gentle	Slow	Fast	Total
Grasp	60	21	61	21	22	**185**
Offer	61	20	62	21	21	**185**
Drop	38	21	41	20	20	**140**
Point - Surface	39	20	37	20	20	**136**
Thumb up	38	20	40	20	20	**138**
Silence	19	19	20	20	20	**98**
Point - You	39	21	40	21	18	**139**
Total	**294**	**142**	**301**	**143**	**141**	**1,021**

Bold value indicated the number of repetions for each action type across different vitality forms.

### Technical setup

**Figure 2:**
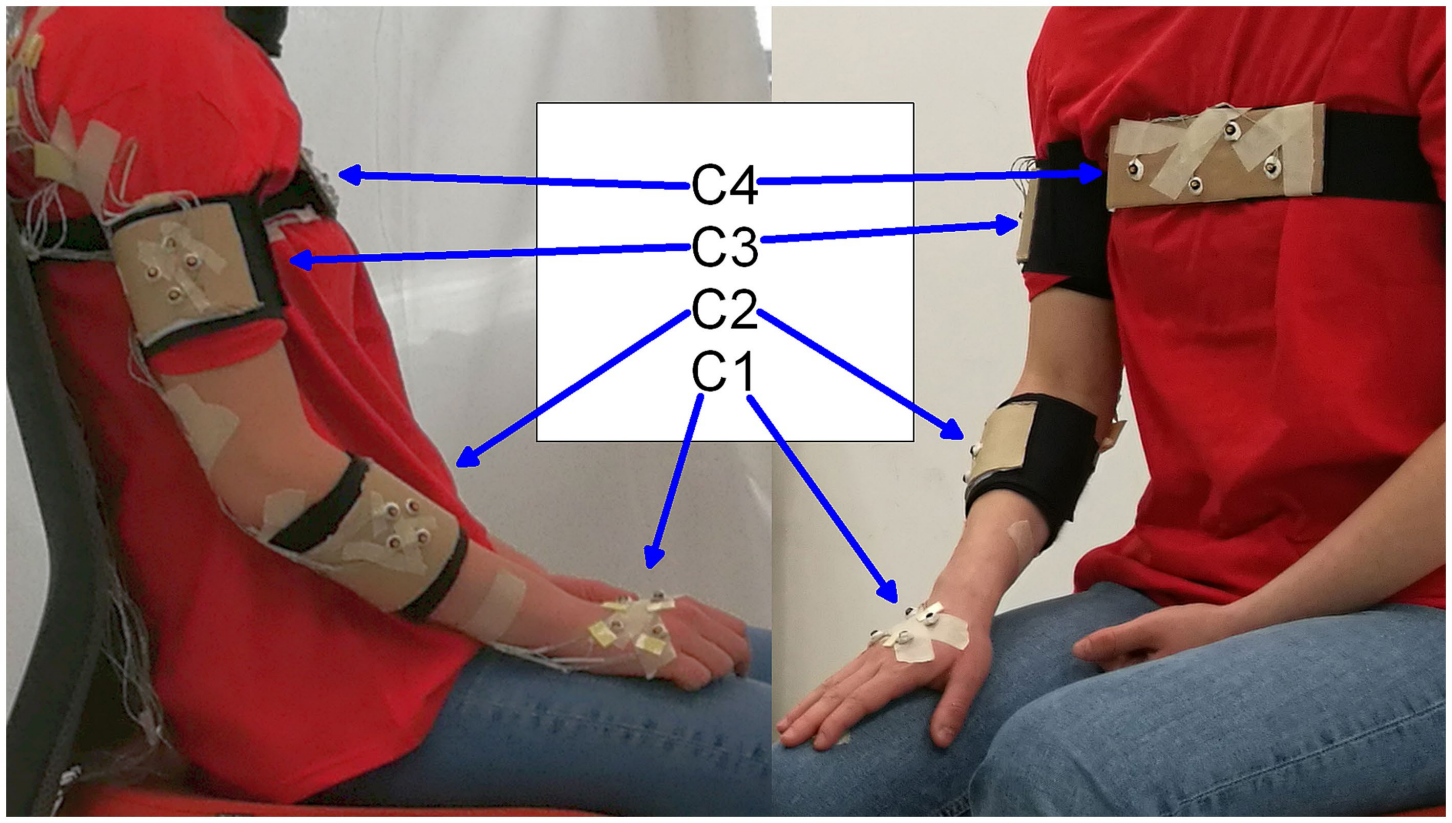
Markers and rigid body positions: C1 – wrist, C2 – elbow, C3 – shoulder, C4 – chest.

The multimodal data were recorded using the NDI Optotrack Certus system, and 2 webcams (one frontal and one lateral). The motion capture system (3D positional data, at 100fps) and the video recording (1920 × 1,080 at 30fps) were synchronized using YARP platform. The synchronized data playback was realized with the freely available EyesWeb software (Volpe et al., 2016). During the execution of each action, the 3D positions of 16 markers were recorded. Specifically, markers were assembled into four rigid bodies placed close to the wrist, elbow, shoulder, and on the torso of each actor (Figure 2). Two additional markers were used to refer to the table placed in front of the actor.

### Data processing

The aim of this analysis was to investigate spatiotemporal cues that characterize VFs. Data preprocessing, feature extraction and statistical analyses were carried out using Matlab software. To reduce the dimensionality of the input data, we computed the centroids of the marker positions on each of 4 locations on the body (Figure 2). We thus obtained four such centroid positions. Then, we focused on kinematic features of the wrist (C1), and positional properties of the elbow (C2), shoulder (C3) and chest (C4). All actions started from the same initial position and ended at approximately the same position. We divided each action into two major phases: GO-phase (from the starting to the ending position) and Return-phase (from the ending position to the starting position). Automatic segmentation of the actions was performed by using preselected velocity and acceleration thresholds. For statistical analysis, we only considered the GO-phase because the actors were instructed to focus only on the goal of the action and not on the Return-phase.

### Feature extraction

We proposed 22 features to assess possible differences among five different action conditions (gentle, neutral, rude, slow, fast). Some of these features referred to specific action moments (e.g., the maximum velocity), whilst others described the whole action (e.g., the suddenness). We explored the existing literature on related topics, such as emotion recognition (Piana et al., 2016) and social relations recognition (Beyan et al., 2017) from full-body movements, and the analysis of the expressive quality of movement (Mazzarino and Mancini, 2009; Niewiadomski et al., 2013, 2015, 2019) to define a set of kinematic features for this study. The following features were extracted:

*(1–3) Velocity Maximum* (Vmx), *Acceleration Maximum* (Amx), and *Jerk Maximum* (Jmx). These are three standard kinematic features that are frequently considered in previous works. For these three features, a highest value was computed over the entire gesture phase, and referred to as maximum. Previous works showed that the velocity can be relevant for the distinction between rude and gentle (Lee et al., 2017). Additionally, the *Vmx* feature was used to verify the data collection design (i.e., control analysis) and more specifically to check whether actions that were intended to be slow, were indeed slower than the neutral ones (i.e., in baseline condition) and whether fast actions were actually quicker than the neutral ones.

*(4) Suddenness* (S) was calculated by adopting the method described in Niewiadomski et al., 2015. In our case, the value of the *Suddenness* was a value of the *α* parameter of the α-stable distribution of the wrist velocity computed on the whole GO-phase. This feature might be relevant, as the rude actions were expected to be more sudden than other actions. It is important to notice that while Vmx referred to one instant of the movement, the *Suddenness* feature described the velocity profile during the whole action.

*(5) Maximum Path Offset Wrist* (POWmx): was defined as the maximum deviation of the trajectory of the wrist from a straight line joining the starting and ending points. This feature measured how curved the trajectory was.

*(6) Maximum Path Offset Shoulder* (POSmx) was the maximum deviation of the trajectory of the shoulder’s centroid from a straight line.

*(7) Percent Maximum Path Offset Wrist* (%POWmx) was the percentage of the gesture duration from its start to the maximum path offset (deviation) of the wrist.

*(8) Percent Maximum Path Offset Shoulder* (%POSmx) was the percentage of the gesture duration from its start to the maximum path offset (deviation) of the shoulder.

*(9) Arc Length* (AL) was the total length of the trajectory from the beginning to the end/goal of the action. It was obtained by calculating the cumulative sum of distances between points on the trajectory.

*(10) Wrist Curvature Maximum* (CWmx) was the maximum trajectory curvature of the wrist.

*(11–13) SMoothness Average* (SMa), *SMoothness Standard deviation* (SMsd), *SMoothness Maximum* (SMmx) measured how smooth the trajectory of the movement was by considering the wrist. These features were calculated according to the procedure described by Mazzarino and Mancini (2009).

*(14–16)* Var*iability Wrist Maximum* (VWmx), *Variability Wrist Average*, (VWa), and *Variability Wrist Standard Deviation* (VWsd) represented the movement variability and namely the difference between the actual wrist velocity and the low pass filtered velocity of the same joint. These features were computed according to the method proposed in Niewiadomski et al., 2019.

*(17–19) Angle Arm-Chest Maximum* (AACmx), *Angle Arm-Chest Average* (AACa) and *Angle Arm-Chest Standard Deviation* (AACsd) measured the maximum, average and standard deviation of the angle between the arm bone and the chest of the actor performing the action.

*(20–22) Angle Elbow Maximum* (AEmx), *Angle Elbow Average* (AEa), *Angle Elbow Standard Deviation* (AEsd) measured the maximum, average and standard deviation of the angle between the arm bone (the humerus) and that of the forearm during the action execution.

### Data analysis and results

**Figure 3:**
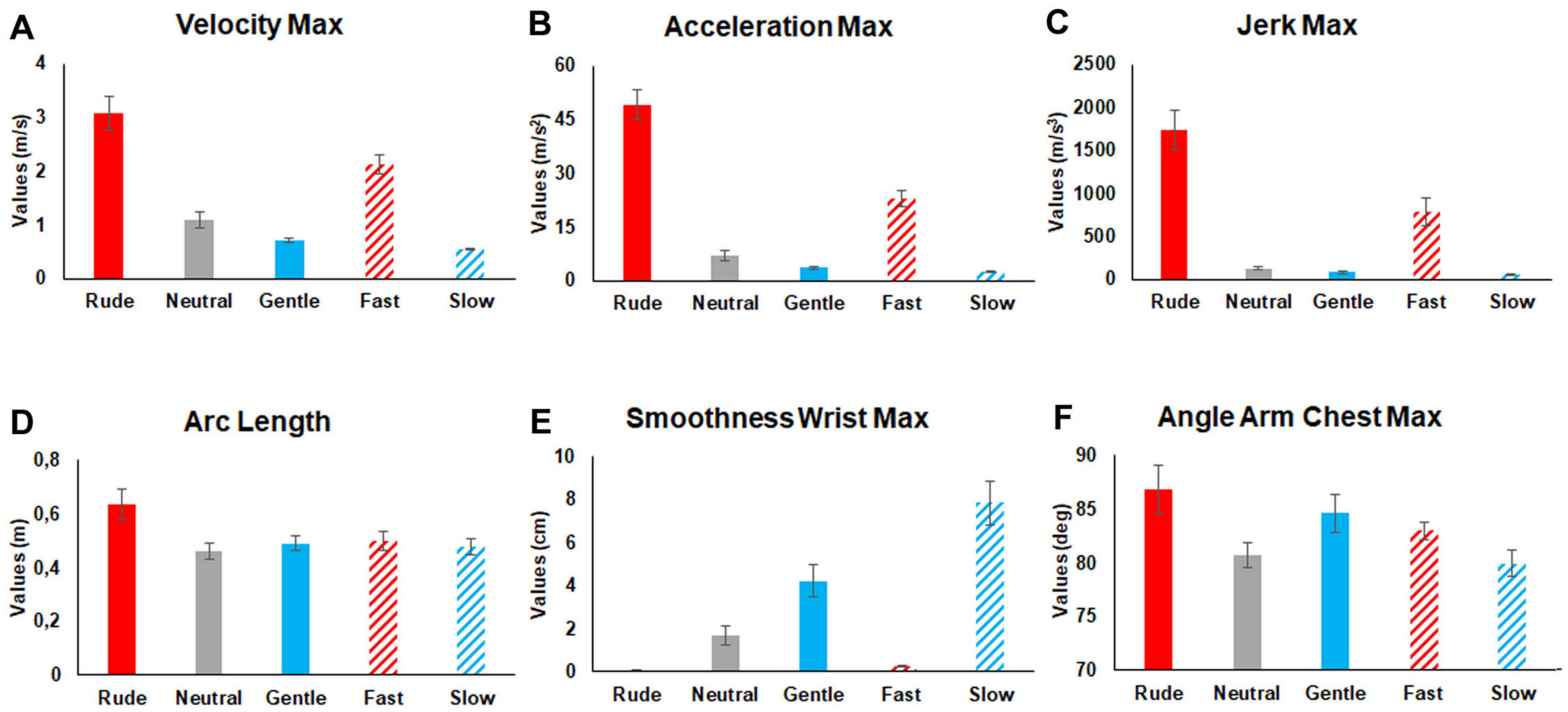
Mean values of the most representative kinematic features characterizing all the actions performed in five different ways (rude, neutral, gentle, fast, slow). Comparison among conditions and related significance values are reported in Table 2.

After the identification of these twenty-two features, we tried to assess their variations during the execution of the seven actions performed in different ways (neutral, gentle, rude, slow, fast). Specifically, our main goal was to understand whether and how these features can be used to differentiate vitality forms. To this purpose, mean values of each feature were modeled using a Repeated Measures General Linear Model (GLM). Outlier values were calculated and discarded from the subsequent statistical analysis (> 2.5 SD of the mean). Sphericity of data was verified before performing statistical analysis (Mauchly’s test, *p* > 0.05) and the Greenhouse–Geisser correction was applied in case of sphericity violation (*p* < 0.05). All variables were normally distributed (Kolmogorov–Smirnov Test, *p* > 0.05). In total 22 Repeated Measure GLM were carried out, one for each feature. The significance level was fixed at 0.05 *p* value. Each GLM comprised the mean value of each feature obtained during the execution of seven actions performed by the two actors (male and female) in different ways (gentle, neutral, rude, slow, fast). Results of the GLM analyses and *post hoc* comparison are reported in Table 2. Mean values of the most representative kinematic features (*Velocity Maximum, Acceleration Maximum, Jerk Maximum, Arc Length, SMoothness Maximum, Angle Arm-Chest Maximum*) characterizing all the actions performed in different ways are shown in Figure 3 (see also Table 2 for statistical results). Notably, to check possible differences between the two actors, the average of each kinematic parameter related to actions performed by the female actress was compared to those performed by the male actor by a pairwise t-test. Results of this analysis indicate a significant difference (*p* < 0.05) for the following kinematic features: Angle Arm-Chest Maximum (AACmx), Angle Arm-Chest Average (AACa), Angle Arm-Chest Standard Deviation (AACsd), Angle Elbow Maximum (AEmx), Angle Elbow Standard Deviation (AEsd), Arc Length, and Percent Maximum Path Offset Shoulder (%POSmx).

**Table 2:** Here are reported the mean values calculated for all the features.

	Gentle (a)	Neutral (b)	Rude (c)	Slow (d)	Fast (e)	F-score	*p*-value
**Vmx**	0.7 **bec**	1 **deac**	3 **bdea**	0.5 **bec**	2.1 **bdac**	73.4	*p* < 0.0001
**Amx**	3.6 **ec**	7 **ec**	49 **bdea**	2.5 **ec**	22.8 **bdac**	128.4	p < 0.0001
**Jmx**	81.4 **ec**	128.8 **ec**	1737.1 **bdea**	47.8 **ec**	788.2 **bdac**	45.3	p < 0.0001
**S**	1.8 **c**	1.3	1.1 **dea**	1.7 **c**	1.6 **c**	5.1	*p* < 0.01
%POSmx	39.7	35.1	38.7	44.7	37.3	2	*p* > 0.05
POSmx	0.1	0.1	0.1	0.1	0.1	1.9	p > 0.05
%POWmx	35.5	33.1	32.5	37.7	39.3	1	p > 0.05
POWmx	0.55	0.08	0.16	0.09	0.15	1.1	p > 0.05
**AL**	0.49 **c**	0.46 **c**	0.6 **bdea**	0.47 **c**	0.4 **c**	14.6	p < 0.0001
**CWmx**	12.5 **c**	28.5 **c**	268.2 **bdea**	7.4 **c**	65.9 **c**	4.5	p < 0.01
**SMmx**	4.2 10^−7^ **bdec**	1.7 10^−7^ **da**	0.07 10^−7^ **da**	7.8 10^−7^ **beac**	0.2 10^−7^ **da**	35.8	p < 0.0001
**SMa**	4.2 10^−7^ **bdec**	1.7 10^−7^ **da**	0.07 10^−7^ **da**	7.8 10^−7^ **beac**	0.2 10^−7^ **da**	36.2	p < 0.0001
**SMsd**	10 10^−15^ **bdec**	2.8 10^−15^ **da**	0.07 10^−15^ **da**	20.1 10^−15^ **beac**	0.2 10^−15^ **da**	50.6	p < 0.0001
**VWmx**	7.8 **c**	8.1 **ec**	35 **bdea**	7.4 **c**	14.4 **bc**	33.5	p < 0.0001
**VWa**	0.05 **ec**	0.12 **ec**	1.1 **bdea**	0.02 **ec**	0.8 **bda**	10	p < 0.0001
**VWsd**	0.6 **ec**	1 **ec**	9.9 **bdea**	0.5 **ec**	4.1 **bdac**	53.3	p < 0.0001
**AACmx**	84.6 **bd**	80.7 **ac**	86.8 **bde**	79.9 **ac**	82.9 **c**	9.9	p < 0.0001
**AACa**	63.3	62.6 **c**	63.8 **bde**	62.5 **c**	62.7 **c**	5.4	*p* < 0.01
**AACsd**	19.1 **bd**	16.2 **ac**	20.6 **bde**	15.9 **ac**	17.6 **c**	9.2	p < 0.0001
**Aemx**	82.4 **be**	80.8 **a**	81.6	81.5	80.7 **a**	4.2	p < 0.01
AEa	72.2	71.5	72.4	70.8	72.2	2	p > 0.05
**AEsd**	13.5 ec	13.2 **ec**	11.4 **bda**	12.9 **ec**	11 **bda**	5.5	p < 0.01

The letters a-e indicate significant differences in pairwise comparisons (GREENHOUSE–GEISSER correction). Features for which results are significant (*p* < 0.05) indicated in bold.

Velocity Maximum (Vmx), Acceleration Maximum (Amx), Jerk Max (Jmx), Suddenness (S), Maximum Path Offset Wrist (POWmx), Maximum Path Offset Shoulder (POSmx) Percent Maximum Path Offset Wrist (%POWmx), Percent Maximum Path Offset Shoulder (%POSmx), Arc Length (AL), Wrist Curvature Maximum (CWmx), SMoothness Average (SMa), SMoothness Standard deviation (SMsd), SMoothness Maximum (SMmx), Variability Wrist Max (VWmx), Variability Wrist Average (VWa), Variability Wrist Standard Deviation (VWsd), Angle Arm-Chest Maximum (AACmx), Elbow- Shoulder-Chest (ESC), Angle Arm-Chest Average (AACa), Angle Arm-Chest Standard Deviation (AACsd), Angle Elbow Maximum (AEmx), Wrist-Elbow-Shoulder (WES), Angle Elbow Average (AEa), Angle Elbow Standard Deviation (AEsd).

Summing up, our results show that considering the standard kinematic features *Velocity Maximum* (Vmx), *Acceleration Maximum* (Amx), and *Jerk Maximum* (Jmx) it is possible to distinguish gentle actions from rude actions (Figure 3). Most interestingly, using other features, it is possible to distinguish rude actions from fast actions, and gentle actions from slow actions.

Concerning rude VFs, the three main features (Vmx, Amx, Jmx) were higher for rude actions than for fast ones, while the overall measure of Suddenness (S) showed that rude actions were more sudden than other ones. Additionally, rude actions also resulted significantly longer (AL) than any other actions, including fast ones. Other significant differences between rude and fast actions were also observed for the variability features (VWmx, VWa, VWsd).

Concerning gentle VFs, the three standard kinematic features (Vmax, Amax, Jmax) did not allow to distinguish between gentle and slow actions. The gentle actions differed from slow ones, mainly considering features based on arm-chest angle (AACmax and AACsd), and on smoothness-based features (SMmx, SMa). Particularly, gentle actions were performed with a significant wide arm-chest angle and a less smoothness in comparison to slow actions.

### Vitality forms classification

In this section, we provided the results of classification analysis carried out for VFs. Standard classifiers were used: (a) SVM with RBF (SVM-RBF), (b) and polynomial kernel (SVM-PL), (c) k-NN, (d) Multi-layer Perception (MLP) and Random Forest (RF) that were widely considered in the past to recognize human internal states from the kinematics data of upper and full body nonverbal behaviors [see, e.g., Piana et al. (2016), Niewiadomski et al. (2016), Fourati and Pelachaud (2015), and Karg et al. (2010)]. To train the classifiers, we used 17 features for which the effect of VFs was observed in GLM analysis (see Table 2). Five classes were considered: gentle, neutral, rude, slow and fast. Before training, normalization was applied to all the data. All 1,021 actions were used for the classification. The classes were not balanced (Table 1).

### Leave-one-out validation

In the first series of experiments, the leave-one-out method was used. Grid research was performed to tune SVM using consecutive powers of 2 in the range 1, 9. The best performance was obtained for *C* = 8 and *γ* = 16. In the case of SVM-PL the best results were obtained for *C* = 8 and *γ* = 8. All the results are described in Table 3 in terms of average accuracy, F-score, Recall and Precision.

**Table 3:** Summary of the results obtained using different classifier algorithms with leave-one-out validation.

Algorithm	Accuracy	F-score	Precision	Recall
SVM-RBF	87.4	87.3	87.4	87.5
SVM-PL	85.4	85.4	85.5	85.4
k-NN	86.3	86.3	86.5	86.3
MLP	81.7	81.6	81.6	81.7
RF	84.9	84.8	84.9	84.9

In all cases, the accuracy was significantly above the chance level (29%). Most of the classifiers performed similarly on the dataset, with SVM-RBF achieving the highest numerical results and MLP performing the worst. Table 4 shows the confusion matrix for SVM-RBF. Although good results in general were obtained, still some confusion was observed. Specifically, gentle actions were quite often confused with the slow ones, but rude actions were only very rarely confused with fast actions.

**Table 4:** Confusion matrix of the SVM-RBF classifier.

	Gentle	Neutral	Rude	Slow	Fast
Gentle	257	15	6	23	0
Neutral	13	123	3	0	3
Rude	1	2	282	0	9
Slow	24	4	9	104	0
Fast	1	1	14	0	127

### Leave-one-gesture-type-out validation

Additionally, we ran a series of computations using the leave one-gesture-type-out procedure. Each time we removed one action type (e.g., all occurrences of grasping or all occurrences of thumb up action) from the training set and we used the data of that action as a test set. This procedure was repeated seven times, and each time dimensions of the training and testing set were different (preserving the total number of 1,021 actions), as the number of trials for each action type was slightly different. This analysis simulated the situation in which the previously trained model was used to recognize VFs of a new gesture (which was not present in the training set). In each computation, independent parameters tuning was performed for SVM-PL, and SVM-RBF. The average results for seven iterations are provided in Table 5.

**Table 5:** Summary of the results obtained considering the leave-one-gesture-type-out validation with different classifier algorithms.

Algorithm	Accuracy %	F-score	Precision	Recall
SVM-RBF	74.4 (5.84)	73.5 (5.76)	76.9 (5.28)	74.4 (5.85)
SVM-PL	74.7 (6.92)	74.2 (6.27)	78.3 (5.37)	74.6 (6.94)
K-NN	63.6 (6.78)	63.2 (7.08)	66.7 (6.22)	63.6 (6.82)
MLP	68.0 (6.13)	67.0 (6.05)	71.4 (5.78)	68.0 (6.15)
RF	72.8 (6.89)	71.5 (7.04)	75.3 (5.80)	72.8 (6.91)

Standard deviation is reported in prenthesis.

Unsurprisingly the overall results in this case were lower than the results reported in Table 3. However, results are still highly above the chance level showing that it is possible to recognize VFs even from the actions which were not present in the training set. Most of the classifiers performed similarly on the dataset. The ANOVA results indicate that there is a statistically significant difference between the F-scores’ means (F-statistic = 3.1126, *p* < 0.05). However, most pairwise comparisons using Tukey HSD are not statistically significant, except for the comparison between SVM-PL and KNN. Additionally, the highest F-score was obtained with SVM-PL.

**Figure 4:**
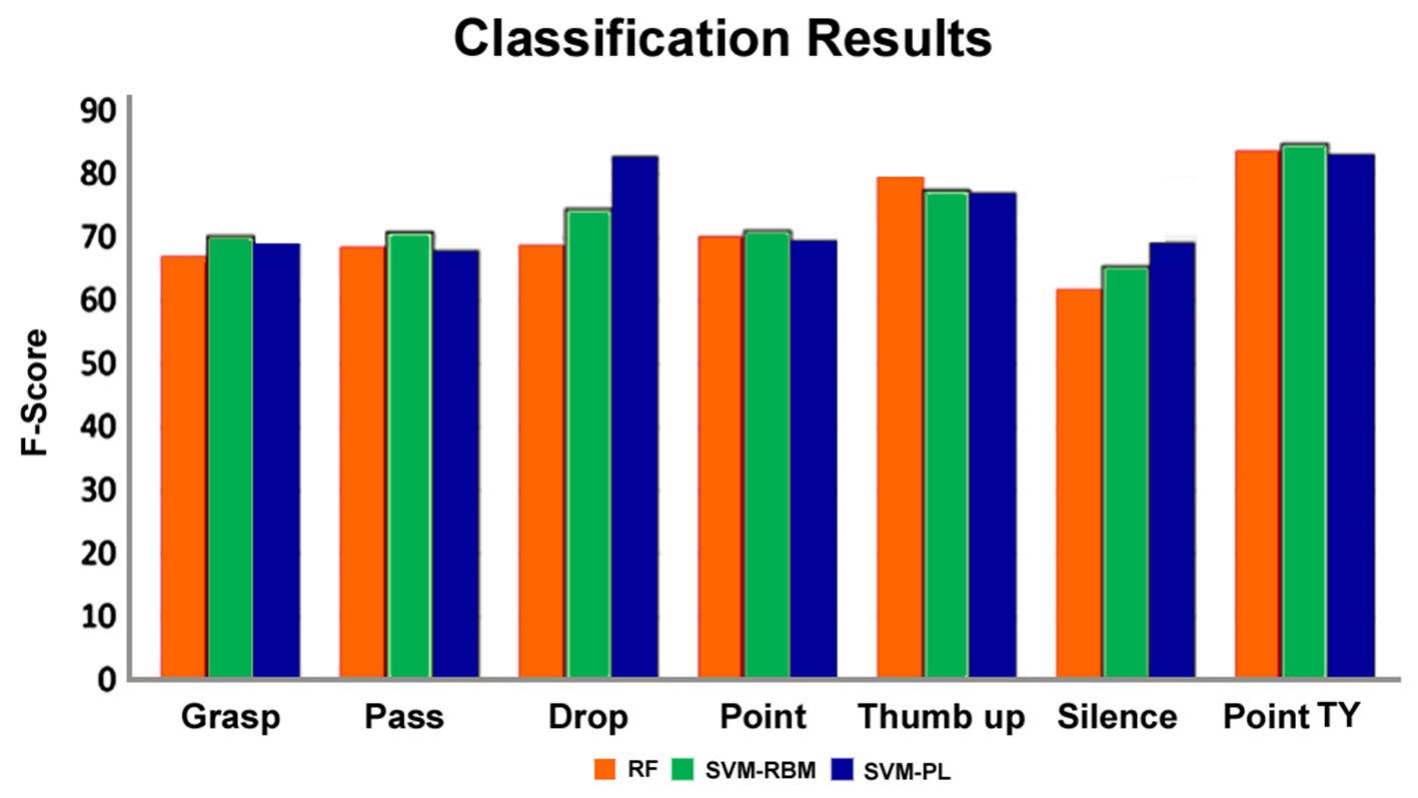
The F-scores related to each type of action when using leave-one-gesture-type-out method and RF, SVM-RBM, and SVM-PL.

When analyzing individual action types, the ANOVA results also indicate a statistically significant difference between the F-scores’ means (F-statistic = 6.94, *p* < 0.05). Again, most pairwise comparisons using Tukey HSD are not statistically significant, except for the comparisons between the “pointing toward a person” gesture and the grasping, passing, pointing to the surface, and silence gestures, as well as between the silence and thumb-up gestures. Additionally, the numerically highest score was obtained for the “pointing toward a person” gesture, while the lowest score was for the silence action (Figure 4). This probably happened because the silence action was quite different from any other action collected in our dataset and may require a specific kinematic modulation of the actor to preserve its meaning.

### Best features identification

To compare the relative relevance of different features in assessing VFs, we performed a set of computations with only one feature at once. The Random Forest was used, for this purpose, as it does not require time-consuming parameter tuning. The best performance was obtained for *SMoothness Standard deviation* (SMsd, Acc = 59.1, F-score = 56.1), which was followed by *Acceleration Maximum* (Amax, Acc = 54.4, F-score = 54.4), *SMoothness Maximum* (SMmx, Acc = 53.8, F-score = 53.8) and *SMoothness Average* (SMa, Acc = 53.8, F-score = 53.8), Var*iability Wrist Standard Deviation* (VWsd, Acc = 53.7, F-score = 54), *Velocity Maximum* (Vmx, Acc = 52.6, F-score = 52.4) and *Jerk Maximum* (Jmx, Acc = 50.7, F-score = 50.5), *Variability Wrist Average* (VWa, Acc = 46.8, F-score = 46.9) and *Variability Wrist Maximum* (VWmx, Acc = 44.5, F-score = 44.6), *Wrist Curvature Maximum* (CWmx, Acc = 32.7, F- score = 33.2), *Arc Length* (AL, Acc = 31.3, F-score = 31.3), *Angle Arm-Chest Standard Deviation* (AACsd, Acc = 28.6, F-score = 28.7) and *Suddenness* (S, Acc = 33.5, F-score = 28.7). It is important to notice that the top 7 features also appear (although in different order) among the features which received the highest *F*-value within the statistical analysis (see Table 2). Regarding these results, although the kinematics properties of the action (Jerk, Acceleration, Velocity) allow to distinguish VFs (gentle from rude), the other features, and in particular, the features measuring smoothness and motion variability are also powerful VFs discriminators. At the same time, one may see the huge difference in terms of accuracy and F-score between the results reported in the above paragraph and Table 3. It seems that the combination of different action features is needed to ensure a good recognition rate. To analyze further this hypothesis, we checked the RF performance on various subsets of the 17 features. In Table 6 we report some interesting results.

**Table 6:** Classification results for pre-selected subsets of features.

Considered features	Accuracy	F-score	Precision	Recall
Amx + SMsd	61.5	61.3	61.3	61.5
Amx + Vmx	62.2	61.8	61.6	62.2
Amx + SMa	64.2	63.8	63.7	64.3
Amx + VWsd	64.4	64.2	64.1	64.4
Amx + SMsd + VWsd	65.7	64.8	65.1	65.7
Amx + Vmx + SMsd	66.6	65.9	65.9	66.6
Amx + SMmx + SMa + SMsd + VWsd	67.7	67.3	67.4	67.8
Amx + Vmx + SMsd + VWsd	71.4	70.8	71.1	71.4

In the last step, we performed Principal Factor Analysis on 17 features (using eigenvalue-one criterion and Varimax rotation). Six main components resulted from this analysis:

- Component 1: Jmx (0.908), VWmx (0.886), Amx (0.868), VWsd (0.837), Vmx (0.644), AL (0.624) which, among others, regroups features related to peak an action;
- Component 2: SMa (0.945), SMmx (0.945), SMsd (0.866) related to smoothness of the wrist movement;
- Component 3: AACm (0.919), AACsd (0.916) - concerns the shoulder angle;
- Component 4: AEsd (0.859), AEmx (0.854) - regards the elbow angle;
- Component 5: AACa (0.953), AL (0.609);
- Component 6: S (−0.737), CWmx (0.662), VWa (−0.509), which principally measures whether the movement contains abrupt changes and irregularities.

When considering six input features (one for each PCA component: Jmx, SMmx, AACsd, AEsd, AACmx, and S), the F-score of classification increases to 82.4 (Precision: 82.4, Recall: 82.6). This is compared to an F-score of 84.8 achieved when all features are used with the same classification method (see Table 2), 54.4 when only the *Acceleration Maximum* (Amx) is used, and 61.8 when Amx is combined with Vmx (see Table 6). These results further confirm that the VFs consist of combination of kinematics and trajectory-related characteristics.

## Discussion

In this paper we investigated which features of a movement can reveal information of VFs and whether it is possible to recognize such VFs automatically. To do so, we generated a novel dataset consisting in a variety of actions executed by two actors, either with rude and gentle VFs or without VFs, but with modulations of speed (neutral, slow and fast actions). The analysis of the kinematic properties of the collected actions demonstrated that significant differences exist between the features characterizing gentle and rude VFs, even when applied to different goal directed actions such as pointing, offering or dropping an object etc. More interestingly, we showed that actions endowed with VFs significantly differ from actions performed with different speeds. In other words, rude actions are different from fast actions, while gentle actions are different from slow actions. A proper differentiation among actions with rude and gentle VFs and actions simply performed at a natural, faster or slower pace can be achieved only considering the combination of several features, including kinematic properties (velocity, jerk) and trajectory-based features measuring the movement smoothness, variability, and angles. Standard kinematic features (e.g., velocity peak) were not sufficient to distinguish gentle and slow movements present in our dataset.

Another fundamental finding of this paper is the first demonstration of the possibility of performing automatic VFs recognition. Our results show that it is possible to classify different VFs, reaching up to 87.3% (F-score). Moreover, the classifiers are able to generalize to actions do not present in the training set, achieving an average F-score of 74.2%. Notably, our classifiers were able to distinguish very well rude actions from fast actions, and, to a lower extent, gentle action from slow actions. Pooling together, results of this study clarify two important points: (a) gentle and rude VFs are characterized by a specific set of kinematic features; (b) using these set of features it is possible to recognize VFs automatically. It is important to note that, since VFs are characterized by a set of features, these data show that modifying only one parameter, such as peak velocity, is not enough to transform a slow or fast action into a gentle or rude one.

To date, it is important to emphasize that all human actions convey two key pieces of information: the action goal (what we are doing) and the action VFs (how we are doing it). This distinction is not merely conceptual, but also anatomical. Specifically, while the goal of an action is processed by the parieto-frontal network, the vitality forms (VFs) are encoded by the insulo-cingulate network (Di Cesare et al., 2024). These networks enable us, during social interactions, to immediately understand both what another individual is doing and their mood or attitude toward us. Most intriguingly, these circuits are also activated when we perform actions directed toward others. This highlights the essential role of VFs in navigating the social world and fostering effective interactions.

In addition to actions, human interactions are deeply shaped by linguistic exchanges. In a previous study, we demonstrated that listening to action verbs pronounced with gentle or rude vitality forms, as well as imagining pronouncing the same verbs with identical VF, activating both the parieto-frontal circuit and the dorso-central sector of the insula (Di Cesare et al., 2017b). This mechanism allows individuals not only to understand the vitality forms expressed by others through speech or actions by remapping these features onto their motor schemas, but also to prepare an appropriate motor response. During social interactions, the affective state of an agent, communicated through vitality forms in speech and actions, plausibly modulates the motor behavior of the recipient. This hypothesis was supported by a kinematic study (Di Cesare et al., 2017a). Specifically, participants were presented with task-related motor requests (e.g., “give me” or “take it”) expressed gently or rudely through visual, auditory, or combined (visual and auditory) modalities. In response to this request, participants were asked to either give or take a bottle. The results showed that VFs of the request (gentle or rude) significantly influenced the execution of the participants’ actions. Participants interacted with the object using a larger trajectory and higher velocity in response to rude requests, while gentle requests led to smaller trajectories and lower velocities, indicating kinder interaction. These findings provide clear evidence that the VFs expressed by an agent can directly impact the motor behavior of the recipient. Interestingly, VFs influence not only the execution of actions but also their perception. Since the perception, planning, and execution of VFs rely on the same neural circuits, a psychophysical study revealed that vocal VFs affect the duration of the internal representation of subsequent actions (Di Cesare et al., 2021). Particularly, participants listened to an actor or actress voice expressing a motor request (e.g., “give me”) with either gentle or rude vitality. Afterward, they observed task related video clips showing only the initial phase of a gentle or rude passing action and were asked to mentally simulate the action’s continuation, indicating when it would end. The results showed that listening to a gentle vocal request increased the estimated duration of the subsequent action, whereas listening to a rude vocal request decreased it. These findings demonstrate that interacting with an agent expressing task-related requests conveying VFs modulates the motor behavior of the recipient, highlighting their critical role in social interactions. Very recently, Lombardi et al. (2024) demonstrated that, when people interacted with a humanoid partner such as the iCub robot, its facial expression significantly modulated some kinematic parameters characterizing their actions. Most interestingly, the observation of positive or negative humanoid facial expressions modified the perception of VFs and influenced the interaction of participants toward the iCub robot. Specifically, if the iCub robot performed a rude request with a happy facial expression, compared to the same request with an angry facial expression, participants perceived it as communicating a positive attitude and interacted with the robot by decreasing their velocity response. In contrast, if the iCub robot performed a gentle request with an angry facial expression, compared to the same request with a happy facial expression, participants perceived it as communicating a negative attitude and thus interacted with the robot by increasing their velocity response. Pooling together, these findings lay the foundation for a future in which humanoid robots could accurately detect and interpret action VFs and facial expressions, enabling them to better understand human partners and plan appropriate responses toward them.

Pooling together, all these findings demonstrated that VFs expressed by a human or robotic partner influence the perception and the motor response of the receiver highlighting the VFs relevance in social interactions. However, a compelling question remains: how is this mechanism modulated? Some possible factors may be involved in this modulation effect such as the social scenarios (i.e., at dinner, at work etc.), the role of the individual in that social context (passive or active), the congruence between the perceived action VFs (i.e., I ask you that object: give me!) and the subsequent motor behavior (you pass me it).

When considering the social scenarios, it is important to keep in mind that different scenarios may reduce the modulation effect of VFs. For example, at a gala dinner, the response to a rude action might be tempered by the presence of other people around. Furthermore, if we consider the role of an individual in the social context, the level of involvement—whether actively engaged or passively observing—may influence the impact of VFs on their actions and speech. It is reasonable to hypothesize that being physically engaged in a social interaction enhances the encoding of VFs, promoting a smoother and more mutual exchange of action and speech compared to passive observation.

Finally, considering the congruence of VFs with the action context, it is important to determine whether the modulation effect of VFs on behavior arises independently of the context or is specifically tied to goal-directed actions. For instance, does listening to a sentence with VFs unrelated to the action context produce the same modulation effect? It is plausible that VFs exert a stronger influence when they are directly related to the ongoing task. This hypothesis aligns with studies by Mirabella and colleagues (Mancini et al., 2022; Calbi et al., 2022; Mirabella et al., 2023; Montalti and Mirabella, 2023), which showed that during attentional tasks, only information relevant to the task influences participants’ response.

VFs represent a very novel and intriguing research field, and several future experiments will be essential to better understand this action property. For example, as discussed above, VFs performed by an agent influence the motor response of the receiver indicating an automatic motor contagion. Despite the importance of VFs, we know very little about them. In this view, the present study represents an important step to the knowledge of the kinematic features of VFs. In the future, results of this research could have an impact in different fields ranging from neuroscience to social humanoid robotics and virtual agents’ community. For example, it may enable artificial agents to recognize human attitudes and properly adapt their behavior to the partner’s state during interactions, e.g., to communicate politeness (Niewiadomski and Pelachaud, 2010). Such artificial agents endowed with a capacity to generate and understand VFs could be useful in several scenarios such as assistance of elderly people and hospital care (Hammer et al., 2016; D’Incà et al., 2023), serious games and social skills training (Vannini et al., 2011), entertainment (e.g., video games), security and surveillance (Inbar and Meyer, 2019) and customer services. Finally, understanding the features characterizing VFs could be very useful in future to develop a rehabilitative intervention for pathologies characterized by social and communicative disorders such as autism (Daniel et al., 2022; Rochat and Gallese, 2022).

## Limitations

Although our dataset was quite big, all the actions were performed only by two actors. This did not allow us to study interpersonal differences. To deeply understand the relation between VFs expression and kinematic properties, it will be essential in the future to collect more complex actions including other body parts while are performed by numerous individuals. Moreover, while this study focused only on gentle and rude VFs, this approach could be considered restrictive, as people perform various types of VFs in everyday interactions. Future work will focus on increasing the number of VFs to investigate (i.e., annoyed, enthusiastic, calm, disapproved etc.). Finally, the data analyzed in this study were recorded in a laboratory context, which could have affected the expression of VFs. Thus, we plan to improve our approach to record in future VFs in ecological settings and including various social contexts.

## Data Availability

The raw data supporting the conclusions of this article will be made available by the authors upon request via mail.
