# Corrigendum: Decoding communicative action vitality forms in social contexts

**DOI:** 10.3389/fpsyg.2025.1593344

**Published:** 2025-03-26

**Authors:** Radoslaw Niewiadomski, Amrita Suresh, Alessandra Sciutti, Giuseppe Di Cesare

**Affiliations:** ^1^Department of Informatics, Bioengineering, Robotics, and Systems Engineering, University of Genoa, Genoa, Italy; ^2^Cognitive Architecture for Collaborative Technologies Unit, Italian Institute of Technology, Genoa, Italy; ^3^Robotics Research Group, Faculty of Mathematics and Computer Science, University of Bremen, Bremen, Germany; ^4^Department of Medicine and Surgery, University of Parma, Parma, Italy

**Keywords:** social interaction, action - investigation, human interactions, vitality affects, affective communication

In the published article, there was an error in the Funding statement. The name of the funding project “Artificial Intelligence Research (FAIR), funded by the European Union – NextGenerationEU, CUP J33C24000430007” was incomplete.

Original Funding statement:

The author(s) declare that financial support was received for the research, authorship, and/or publication of this article. This work was supported by the European Research Council (ERC) under the European Union's Horizon 2020 Research and Innovation Program. wHiSPER, G.A. No. 804388 to Alessandra Sciutti; National Recovery and Resilience Plan, Call for tender No. 247 of 19/08/2022 and No. 367 of 07/10/2022 funded by the European Union, Project No. SOE_0000049 and Fondazione Cariplo e Fondazione CDP Grant - Supporting Young Italian Talents in the European Research Council Competitions (G.A. No. 2023-2315) to Giuseppe Di Cesare; Artificial Intelligence Research (FAIR), funded by the European Union – NextGenerationEU, CUP J33C24000430007 to Radoslaw Niewiadomski.

The correct Funding statement appears below.

The authors declare that financial support was received for the research and/or publication of this article. This work was supported by the European Research Council (ERC) under the European Union's Horizon 2020 Research and Innovation Program. wHiSPER, G.A. No. 804388 to Alessandra Sciutti; National Recovery and Resilience Plan, Call for tender No. 247 of 19/08/2022 and No. 367 of 07/10/2022 funded by the European Union, Project No. SOE 0000049 and Fondazione Cariplo e Fondazione CDP Grant - Supporting Young Italian Talents in the European Research Council Competitions (G.A. No. 2023-2315) to Giuseppe Di Cesare; PNRR MUR Project PE0000013 Future Artificial Intelligence Research (FAIR), funded by the European Union - NextGenerationEU, CUP J33C24000430007 to Radoslaw Niewiadomski and Alessandra Sciutti.

The authors apologize for this error and state that this does not change the scientific conclusions of the article in any way. The original article has been updated.

